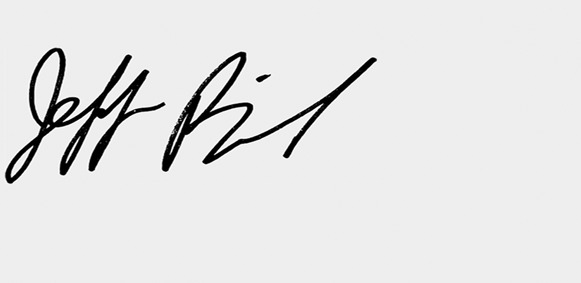# Continuing Growth

**DOI:** 10.5435/JAAOSGlobal-D-20-00013

**Published:** 2020-03-05

**Authors:** Jeffrey S. Fischgrund

**Affiliations:** Dr. Fischgrund is an Editor, Research of the Journal of the American Academy of Orthopaedic Surgeons, Rosemont, IL, and the Professor and Chairman at Beaumont Hospital, Royal Oak, MI.

**Figure:**
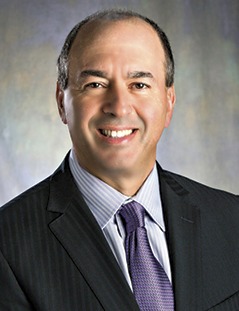
Jeffrey S. Fischgrund, MD

As we look back on the success of the past year and ahead into 2020, I would like to give a big thank you to all of our JAAOS Global reviewers for their support and hard work in 2019 to maintain the quality of *JAAOS Global* at the highest standards. I would especially like to highlight the work of David A. Ajibade, MD, and Thomas A. DeCoster, MD, who have completed 10 or more reviews this past year and recognize Drs. Jad G. Khalil, Ryan James Warth, Rhichard J. Haynes, and Curtis Todd Adams for completing five to nine reviews for JAAOS Global this past year. (see list at http://links.lww.com/JG9/A67).

In addition, I would like to thank Samuel Pantoja, MD, for his work as a deputy editor for *JAAOS Global* and wish him well as he concludes his position as a deputy editor for *JAAOS Global*, and I would like to welcome Julio Urrutia, MD, a new deputy editor. Dr. Urrutia is a titular professor in the Department Traumatología y ortopedia at Pontificia Universidad Católica de Chile.

I am pleased to report that the submissions to JAAOS Global have increased by 65.1% from 2018 to 2019, and we published 28.8% more articles in 2019 compared with 2018. We are very excited to see so much engagement from the submitters and encourage people to continue to submit and engage with JAAOS Global.

JAAOS Global Research and Reviews is now indexed in PubMed Central, Emerging Sources Citation Index (ESCI), the Directory of Open Access Journals (DOAJ), and has been accepted for indexing in Scopus.